# Influences on the physical activity behaviour of inpatients after stroke outside of staff-led rehabilitation sessions: a systematic review

**DOI:** 10.1177/02692155241293269

**Published:** 2024-11-10

**Authors:** Peter Hartley, Katie Bond, Rachel Dance, Isla Kuhn, Joanne McPeake, Faye Forsyth

**Affiliations:** 1 Department of Physiotherapy, 2153 Cambridge University Hospitals NHS Foundation Trust, Cambridge, UK; 2 The Healthcare Improvement Studies Institute, 2152 University of Cambridge, Cambridge, UK; 3 Integrated Therapies Department, 3603 James Paget University Hospitals NHS Foundation Trust, Great Yarmouth, UK; 4 Medical Library, 2152 University of Cambridge, Cambridge, UK; 5 Department of Public Health and Primary Care, 2152 University of Cambridge, Cambridge, UK

**Keywords:** Physical activity, stroke, hospital, COM-B, Theoretical Domains Framework

## Abstract

**Objective:**

To use behavioural science frameworks to synthesise evidence on the factors influencing physical activity of patients hospitalised after stroke outside of staff-led rehabilitation sessions.

**Data Sources:**

A systematic review of qualitative and mixed-methods studies. MEDLINE, PsycINFO, CINAHL, and AMED were searched from inception to October 2024 for studies that explored influences on the physical activity of patients hospitalised after stroke.

**Review Methods:**

Data were coded with reference to the Theoretical Domains Framework and the COM-B (‘capability’, ‘opportunity’, ‘motivation’ and ‘behaviour’) model. Thematic analysis was used to group data extracts into themes within each Theoretical Domains Framework domain. Risk of bias was assessed using the Mixed Methods Appraisal Tool.

**Results:**

We identified 17 studies. There was no significant risk of bias concerns. We identified 19 themes across eight Theoretical Domains Framework domains and all COM-B model categories. The most frequently recognised themes were found in three Theoretical Domains Framework domains: Environmental Context and Resources (themes: 1 ­– availability of sufficient skilled staff to facilitate physical activity; 2 – design and use of the physical environment; 3 – lack of opportunities or incentives; 4 – passivity and institutionalisation; 5 – perceived and actual rules and culture of the ward); Skills (theme: physical impairments); and Social Influences (theme: activity influenced by family and friends).

**Conclusions:**

The review highlights the complexity of the influences on the physical activity of patients hospitalised after stroke outside of staff-led rehabilitation sessions. It is likely multi-component interventions addressing a number of influences will be required to effectively improve physical activity. PROSPERO ID: CRD42022383506.

## Background

In the sub-acute phase after a stroke, a higher dose of physical activity is associated with sustained rehabilitation gains,^1^ and there is a strong dose–response relationship between therapy and physical recovery in the sub-acute and chronic phases of stroke.^2^ The 2023 National Clinical Guideline for Stroke for the UK and Ireland recommends that: ‘people undergoing rehabilitation after a stroke should be supported to remain active for up to 6 hours a day’; this may include up to three hours of activity that is outside of therapist-delivered therapy.^3^ Yet in the UK and internationally, objectively measured levels of physical activity in hospital after stroke are consistently shown to be low, especially outside of therapist-delivered sessions.^4^

**Figure 1.**
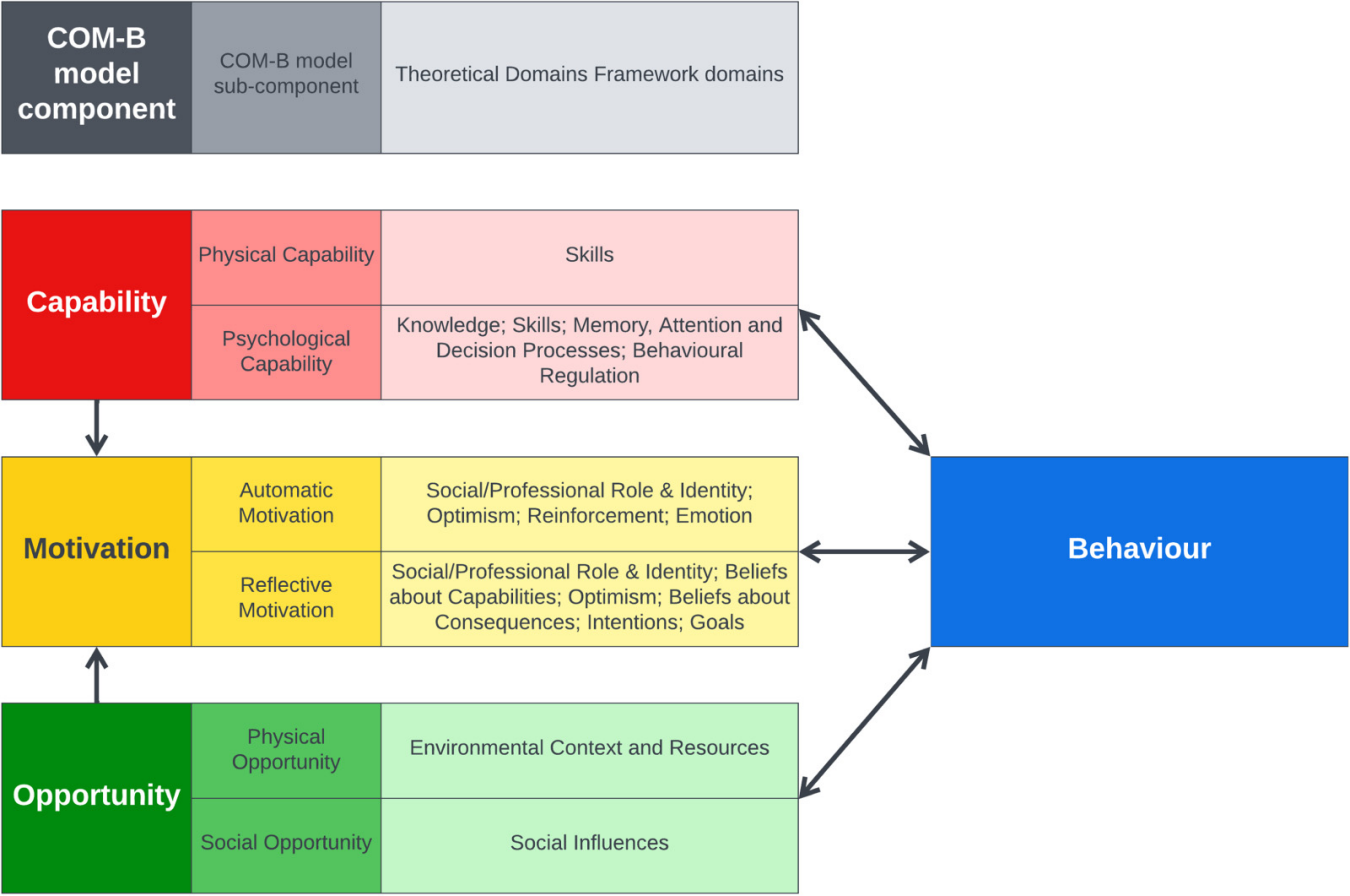
COM-B model and Theoretical Domains Framework.^9,12^

Increasing physical activity in hospital has been shown to be challenging.^5–7^ The development of complex interventions to increase physical activity in hospital may benefit from the use of behaviour change theory.^8^ Behavioural influences can be categorised using various models including the COM-B model^9^ and Theoretical Domains Framework.^10,11^ The COM-B model describes a behaviour in terms of three categories (six sub-categories) that are necessary for the behaviour to be performed: capability (physical and psychological), opportunity (physical and social), and motivation (reflective and automatic).^9^ The Theoretical Domains Framework further defines these categories into 14 domains which can be mapped onto the COM-B model (Figure 1).^10,11^

The Behaviour Change Wheel supports intervention development by mapping the behavioural influences that have been identified to different intervention options that should affect the influences, and thus achieve the desired behavioural change.^9^ Therefore, an understanding of the influences on physical activity behaviour in hospital after stroke would allow for the development of interventions that are targeted at influencing factors that may be amenable to change. The aim of this review is to use the COM-B and Theoretical Domains Framework to synthesise current evidence on the influences on the physical activity of patients in hospitals after stroke outside of staff-led rehabilitation sessions.

## Methods

A protocol for this review was registered on PROSPERO: CRD42022383506 (https://www.crd.york.ac.uk/prospero). The review has been reported in keeping with the Preferred Reporting Items for Systematic Reviews and Meta-Analyses (PRISMA) statement.^13^

The following databases were searched electronically between inception and 6 December 2022: MEDLINE via OVID; PsycINFO via EBSCOhost; Allied and Complementary Medicine Database (AMED) via OVID; CINAHL via EBSCOhost. Updated searches were completed on 16 January 2024 and 2 October 2024. The search strategy is presented in full in the online supplementary material. If a conference abstract was identified that appeared relevant, searches were made to identify a full paper; if no paper was found the abstract was excluded. All reference lists of included studies were searched for other potentially relevant studies missed by the electronic search of databases.

The target behaviour of interest is the physical activity of patients in the hospital. The definition of physical activity includes any bodily movement produced by skeletal muscles that requires energy expenditure.^14^ Included studies had to refer to physical activity. Studies that included analyses of staff-led rehabilitation sessions were included if they also reported on physical activity outside of staff-led rehabilitation sessions. We excluded studies that only evaluated physical activity during staff-led sessions such as physiotherapy sessions.

We included qualitative and mixed-methods studies that explored influences on physical activity behaviour. We included post-implementation evaluations of interventions if the evaluation of physical activity was not specific to a particular activity or exercise. In addition, we excluded papers only available as an abstract or conference proceedings and non-English language studies.

We included studies that examined the physical activity of adults (18 + years) admitted to hospital with an acute stroke. Data on influencing factors could be gathered from various sources, including observations or interviews with patients, their families or visitors, and hospital ward staff. We excluded studies that evaluated the physical activity of samples, who on average were beyond the subacute phase (i.e., with an average time since stroke of more than 12 weeks) of rehabilitation.

The settings of included studies were inpatient hospital wards, either acute stroke wards (including specialist stroke high dependency units), or inpatient rehabilitation wards. Studies that included critical care units were excluded.

Two reviewers independently examined all titles and abstracts by using the pre-defined eligibility criteria. If a reason for exclusion was not evident, the full manuscript was obtained. Full manuscripts of all the studies that remained after title and abstract screening were subsequently examined independently by two reviewers. Disagreements were resolved through discussion with a third author.

Descriptive data for each included study was extracted by one reviewer and checked by a second. Descriptive data included: study design, aim, setting, methodology, sample size, and participant characteristics (i.e., time since stroke or staff disciplines).

Two reviewers independently extracted details related to physical activity behaviour. Where it was not clear whether quotes or statements within included studies referred to physical activity as opposed to social or cognitive activity, the quotes or statements were not included in the analysis.

The risk of bias was assessed using the Mixed Methods Appraisal Tool.^15^ If the analysis plans of mixed-methods studies included the integration of qualitative and quantitative data on influences on physical activity, the mixed-methods tool was used; otherwise, the qualitative tool was used. If a study employed mixed methods for additional reasons to investigate the influences on physical activity and only utilised qualitative data for the investigation of influences on physical activity, the qualitative tool was used, regardless of additional quantitative methods not relating to the aims of this review.

Data synthesis aimed to identify themes in the included studies that describe behavioural influences, and organise themes according to the domains of the Theoretical Domains Framework. Each domain could have multiple or no themes. To achieve this, first, two reviewers independently coded each paper using a deductive approach; that is, data were coded with reference to the Theoretical Domains Framework and COM-B model,^10,11^ using NVivo software. After coding independently, results were exported to Google Sheets software and merged. The two authors reviewed all data coded under each domain of the Theoretical Domains Framework. Each domain was discussed, and all data were checked for consistency and to resolve any differences. At this point, the two reviewers interrogated the data to determine whether there was agreement that the code excerpts related to physical activity (e.g., as opposed to social activity). Where quotes were coded to more than one Theoretical Domains Framework domain, a final decision was made as to which domain and component the data best reflected. Throughout this process, if disagreements were not readily resolved, a third author was asked to contribute their opinions. Final agreement was then made between all three authors. Thematic analysis^16^ was then used to inductively analyse data within each Theoretical Domains Framework domain, to group data extracts into themes. Themes could have a positive, negative, or mixed impact on patient physical activity. Although the COM-B model was not used to categorise themes directly, in certain situations, reviewers referred to its category and subcategory definitions to help select the most appropriate domain within the Theoretical Domains Framework. Specifically, when a theme could be classified under two different domains of the Theoretical Domains Framework, each corresponding to different COM-B subcategories, reviewers took into account the COM-B model's definitions to guide their final decision. The number of studies in which each theme was identified was counted to provide a measure of generalisability.

## Results

**Figure 2.**
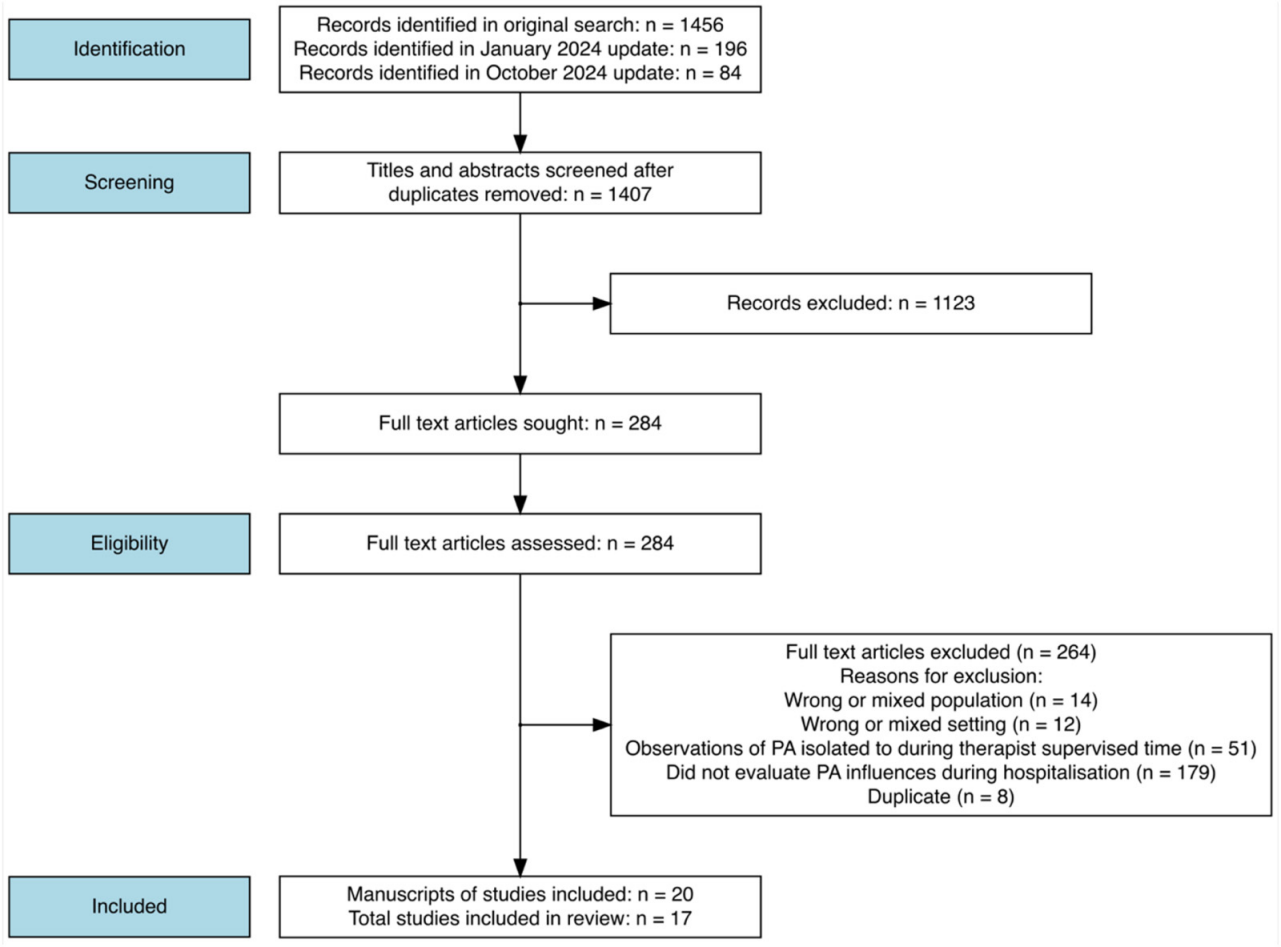
Study flow diagram.

The search identified 20 studies^5,17–35^ (Figure 2). For the purpose of counting the frequency of themes across different studies, certain manuscripts were grouped together as they predominantly used the same data from the same participants, meaning the maximum frequency a theme could be recognised in different studies was 17. Three publications referred to the same programme of work, the Collaborative Rehabilitation in Acute Stroke (CREATE) study and were treated as a single study.^5,19,23^ The study by Kenah, Tavener^32^ reported a secondary analysis of data presented in Janssen, Bird,^21^ including an extra 25 participants.

Descriptions of studies are provided in Table 1. Five studies used mixed-methods,^17,18,23,26,30,33^ 10 studies employed qualitative methods.^20–22,24–29,31,32,34,35^ Morton, Hall^26^ examined behaviour across both inpatient and community settings, though we extracted data only related to the inpatient setting.

**Table 1.** Study characteristics.

Study	Aim	Setting	Data type and collection period	Analysis methods	Patient and carer participants	Patient time since stroke	Staff participants	Staff disciplines
Anaker, von Koch^17^ Sweden Mixed-methods study	To explore and compare the impact of the physical environment on patients’ activities and care at three newly built stroke units.	3 stroke units.	Behavioural mapping and field notes. April 2013 to December 2015.	Deductive content analysis.	55 patients.	Site 1 (median [IQR]): 6.0 (12.3) days; Site 2: 2.0 (1.0) days; Site 3: 9.5 (20.5) days.	n/a	n/a
Clarke and Holt^18^ United Kingdom Mixed-methods study	To identify and explore the perspectives of nurses and other multidisciplinary stroke team members on nurses’ practice in stroke rehabilitation.	2 hyper-acute stroke units; 1 unit with 2 hyper-acute beds and 27 rehabilitation beds; 1 rehabilitation unit.	Q-sorts: October to December 2011; semi-structured interviews: February to May 2012.	Q-factor analysis of q-sort data; directed content analysis of semi-structured interviews.	n/a	n/a	Q-sort: *n* = 65; semi-structured interviews: *n* = 27 (all but 1 also completed Q-sort).	Nurses, HCAs, AHPs, AHP assistants, physicians, carer support officer.
Eng, Brauer^20^ Australia Qualitative study	To explore factors affecting the ability of the stroke survivor to drive their own recovery outside of therapy during inpatient rehabilitation.	2 general rehabilitation wards.	Interviews (with patients and carers); focus group (with staff).	Thematic analysis.	7 patients, 6 carers.	Mean (±SD): 28 (±37) days.	20	Nurses, AHPs.
Janssen, Bird^21^ Australia Qualitative study	To investigate stroke survivors’ perceptions of factors inﬂuencing their engagement in activity outside of dedicated therapy sessions during inpatient rehabilitation.	4 metropolitan rehabilitation units.	Semi-structured interviews. February 2015 to May 2016.	Thematic analysis.	33 patients.	Not stated (within 4 weeks of stroke).	n/a	n/a
Kenah, Tavener^32^ Australia Qualitative study – secondary analysis of Janssen, Bird^21^	To explore stroke survivors’ experiences of non-therapy time to better understand what can lead to people becoming bored.	4 metropolitan rehabilitation units.	Semi-structured interviews. February 2015 to June 2018.	Hybrid approach of inductive and deductive coding and reflexive process of thematic analysis.	58 patients.	Not stated (within 4 weeks of stroke).	n/a	n/a
Janssen, Bird^22^ Australia Qualitative study	To investigate rehabilitation staff perceptions of factors influencing stroke survivor activity outside of dedicated therapy time for the purpose of supporting successful translation of activity promoting interventions in a rehabilitation unit.	4 metropolitan rehabilitation units.	Semi-structured interviews. February 2015 to May 2016.	Stepped iterative process of inductive analysis.	n/a	n/a	Interviews: *n* = 22.	Nurses, AHPs, physicians, social worker.
Jones, Gombert-Waldron,^23^ results also presented in: Jones, Gombert,^5^ Costa, Jones^19^ Donetto, Jones^36^ United Kingdom Mixed-methods study pre and post intervention implementation	To evaluate the feasibility and impact of patients, carers and staff collaborating to develop and implement changes to increase supervised and independent therapeutic patient activity on acute stroke units.	4 stroke units (acute and rehabilitation).	Only pre-intervention implementation semi-structured interviews and ethnographic non-participant observations, data extracted for systematic review. 2016 to 2018.	Thematic analysis.	Interviews = 86 patients Behaviour mapping = 38 patients.	Not stated	Interviews: *n* = 41.	Nurses, AHPs, physicians, psychologists, support worker.
Lipson-Smith, Zeeman^33^ Australia Mixed-methods study	To explore, from the patient perspective, the role of the physical environment in factors crucial to stroke recovery, namely, stroke survivor activity (physical, cognitive, social), sleep, emotional well-being, and safety.	Two inpatient rehabilitation facilities.	Semi-structured interviews, behavioural mapping, questionnaires, and retrospective audit. September 2018 to June 2019.	Interviews analysed using generic qualitative inquiry; quantitative data summarised descriptively. Convergent mixed-methods analysis used to synthesise qualitative and quantitative findings.	36 patients.	Site 1: mean of 36.2 (± 25.5) days; Site 2: mean of 34.3 (± 27.2) days.	n/a	n/a
Loft, Martinsen^24^ Denmark Qualitative study	To describe patients’ experiences with inpatient stroke rehabilitation and their perception of nurses’ and nurse assistants’ roles and functions during hospitalisation.	Stroke rehabilitation unit.	Semi-structured interviews. Collection period not stated.	Content analysis.	10 patients.	Median 4 (range: 3–12) weeks.	n/a	n/a
Maclean, Pound^25^ United Kingdom Qualitative study	To use interviews and non-participant observations to explore perspectives, capability, opportunity and motivation of staff to support stroke survivors to reduce sedentary behaviour.	Stroke unit.	Semi-structured interviews. Collection period not stated.	Content analysis.	22 patients.	Average of 6 weeks.	n/a	n/a
Morton, Hall^26^ United Kingdom Qualitative study	To explore sedentary behaviour after stroke from the perspective of stroke service staff.	2 stroke units (1 rehabilitation, 1 mixed acute/ rehabilitation) with links to community stroke service.	Non-participant observations and staff interviews. Only data relating to inpatients extracted. October 2017 to June 2018.	Thematic analysis.	n/a	n/a	93 patients (non-participant observations); 31 patients (also participated in interviews).	Nurses, HCAs, nursing students, AHPs, AHP assistants, AHP students, physicians, volunteer.
Purcell, Scott^27^ Australia Qualitative study	To explore stroke survivors’ experiences of engagement in occupations during stroke rehabilitation.	Acute stroke and rehabilitation unit.	Semi-structured interviews. Collection period not stated.	Descriptive phenomenological analysis	8 patients.	Mean 25.5 (± 12.1) days.		
Reinholdsson, Herranen^35^ Sweeden Qualitative study	To explore patient experiences of physical activity and inactivity in the stroke unit	9 stroke units.	Semi-structured interviews. September and October 2022.	Thematic analysis	16 patients	Not stated	n/a	n/a
Rosbergen, Brauer^28^ Australia Qualitative study	To understand perceptions and experiences of nursing and allied health professionals involved in implementing an enriched environment in an acute stroke unit.	Acute stroke unit.	Semi-structured interviews. Collection period not stated.	Thematic content approach.	n/a	n/a	10 staff.	Nurses and AHPs.
Simpson, Jose^29^ Australia Qualitative study	To understand from the perspective of stroke survivors and their carers (1) factors contributing to sedentary time and physical activity during inpatient rehabilitation and the transition home, and (2) actual and perceived opportunities to reduce sedentary time and increase physical activity.	3 general rehabilitation units, 2 in public health service, 1 in a private rehabilitation facility.	Semi-structured interviews. August 2018 to May 2019.	Constant comparative process.	7 patient/carer dyads and 8 patients.	Length of rehabilitation admission ranged from 10 days to 90 days.	n/a	n/a
Stewart, Power^30^ Australia Mixed methods study	To describe steps in the design of a participatory, theoretically tailored staff behaviour change intervention to help staff use strategies to increase active practice by stroke inpatients.	Acute stroke and rehabilitation unit.	Focus groups and behavioural mapping. Collection period not stated.	Qualitative framework analysis.	Behaviour mapping = 38 patients.	Not stated.	Focus groups = 21 staff	Nurses, AHPs and physicians.
White, Alborough^31^ Australia Qualitative study	To qualitatively explore the experiences of nursing staff involved in a pilot study investigating the feasibility of an enriched environment in a rehabilitation ward.	Rehabilitation hospital.	Semi-structured interviews. Collection period not stated.	Inductive thematic analysis.	n/a	n/a	22	Nurses
White, Bartley^34^ Australia Qualitative study	To qualitatively explore stroke survivors’ experience of implementation of exposure to an enriched environment within a typical stroke rehabilitation setting, in order to identify facilitators and barriers to participation.	Rehabilitation hospital.	Semi-structured interviews. Collection period not stated.	Inductive thematic analysis.	10 patients.	Between 27 and 65 days in rehabilitation.	n/a	n/a

AHPs = Allied Health Professionals; HCAs = Healthcare assistants, IQR = inter-quartile range, SD = standard deviation.

Risk of bias as assessed by the Mixed Methods Appraisal Tool^15^ is summarised in the online supplementary material. Although both Morton, Hall^26^ and Jones, Gombert-Waldron^23^ used mixed methods as part of their wider project aims of developing interventions, for the purposes of this review, only the qualitative data relating to physical activity influences was extracted. As a result, only the qualitative questions of the Mixed Methods Appraisal Tool were assessed. There were no significant concerns with risk of bias scoring. For the qualitative methodological quality criteria, only three studies had domains scored as ‘can’t tell’ due to insufficient reporting,^20,25,35^ but no other concerns were identified.

We identified 19 themes across eight Theoretical Domains Framework domains and all six sub-categories of the COM-B model. All themes are described in Table 2, within the text we provide more detail on the six themes that were recognised in over half of the included studies. The people whose behaviour was identified in the themes as influencing the patients’ physical activity, included the patients themselves, staff, and family members.

**Table 2.** Description of themes. Themes are organised according to the domains of the Theoretical Domains Framework, which have been further grouped under the categories of the COM-B model, as shown in Figure 1.

Theoretical Domains Framework domains and definitions^11^	Theme and theme description	Example
COM-B: Automatic motivation		
*Emotion* A complex reaction pattern, involving experiential, behavioural, and physiological elements, by which the individual attempts to deal with a personally significant matter or event).	*Grief, loss and low mood* (negative). Number of studies: 3 Low mood and dealing with loss described as affecting patient's motivation with activity and need for ‘down time’.	‘“Depression is a big part … they’re just not going to participate, they just don’t want to … [they have] sort of given up in a way”. (P 5)’ **White, Alborough**^31^
COM-B: Reflective motivation		
*Beliefs about consequences* (Acceptance of the truth, reality, or validity about outcomes of a behaviour in a given situation).	*The patient's belief in whether physical activity and rehabilitation activities will lead to benefits* (positive and negative). Number of studies: 8 patients were more motivated to be physically active when they believed it would benefit them. However, when they didn’t see meaningful benefits, they were less likely to engage. This is closely linked to the domain of goals and the theme of ‘regaining independence and returning home.’ Only one study reported fears of the negative consequences of moving (including falls, worsening symptoms, or COVID-19).^35^	‘Stroke survivors that were able to reframe ‘wasted’ non-therapy time and take charge of their own recovery appeared to be driven by an internal locus of control, a nothing motivates you if you don’t motivate yourself (P11, male, 66 y, moderate stroke) type attitude. Such beliefs were aided by knowledge that activity drives stroke recovery and an understanding of specific exercises and ward activities that they could access. ‘What you do. I mean, you want to get better? You’re not there for a holiday. There is no resort. That's what a lot of people think. You know, bring me food – no, you’ve got to go and get it. Help yourself’. (P34, male, 64 y, mild stroke) this profound sense of agency appeared to be an important personal characteristic in warding off boredom.’ **Kenah, Tavener**^32^
*Goals* (mental representations of outcomes or end states that an individual wants to achieve).	*Goals of regaining degrees of independence and returning home* (positive and negative). Number of studies: 6 Most goals centred on regaining independence, returning to previous lifestyles, or leaving the hospital to go home. These goals generally fostered positive motivation, as participants believed physical activity would help them achieve their aims. However, when patients or staff viewed the goals as unrealistic, it became demotivating.	‘These physical limitations became a motivator for participants to attend therapy, as they saw therapy as the only means of supporting a return to their pre-stroke lifestyles. For some, important events occurring in the near future also provided additional motivation to attend therapy. “the sooner I’m out, the better…. end of September. Yep, that's my goal. It was the… 15 September, but I said I had to be realistic because – see, all our [travel group] is going away … on the 16th. So I wanted to be out by the 15th.” (Agnes, 87 years; FIM 36).’ **Simpson, Jose**^29^
*Social or professional role and identity* (A coherent set of behaviours and displayed personal qualities of an individual in a social or work setting).	*Differences in the approach of different professions towards encouraging and prioritising physical activity* (positive and negative). Number of studies: 4 Therapy staff were viewed as primarily responsible for leading physical rehabilitation and promoting activity, both by patients and staff. This may partly reflect a lack of understanding of nursing staff's roles and their approach to encouraging patient independence.	‘It was clearly stated that the nursing staff often had a different approach to rehabilitation than the therapist. The nursing staff gets to know your goals at the ward rounds, for example. Now I have to practice moving from the chair into the bed, and some of them allow me to. There are also some of them who just “jump over” and lift me into bed. (Patient 8)’ **Loft, Martinsen**^24^
*Knowledge* (An awareness of the existence of something)	*The knowledge and understanding of what the individual can and should be doing, and why* (positive and negative). Number of studies: 7 This was widely seen as a barrier by both patients and visitors. Patients needed clarity on what exercises to do, how to do them, how many, where to exercise (including available equipment), and the reasons for exercising. (Overlap with domain: skills).	‘Patients also felt that communication could enable their activity and wanted more specific ideas from staff, and feedback on what they could do independently or with a carer.’ **Jones, Gombert-Waldron**^23^
COM-B: Physical opportunity		
*Environmental context and resources* (Any circumstance of a person's situation or environment that discourages or encourages the development of skills and abilities, independence, social competence, and adaptive behaviour).	*Availability of sufficient skilled staff to facilitate physical activity* (negative). Number of studies: 13 This theme highlighted the belief that more staff were needed to have sufficient time to support physical activity, as patients depended on staff availability. It also emphasised the need for staff to have the skills and confidence to integrate rehabilitation into routine tasks. This theme closely relates to the idea that other staff duties took precedence over facilitating activity.	‘Weekends were identified as particularly challenging due to the low number of nursing staff on duty, limited medical cover and the absence of therapists. Nursing staff lacked the time to run weekend groups or supervise ward practice, and reported “struggling to get even the basic tasks done like observations and medications”.’ **Stewart, Power**^30^
	*The design and use of the physical environment* (positive and negative). Number of studies: 12 The design, setup, and use of ward spaces, along with the areas available to patients, impacted physical activity both positively and negatively.	‘This contrasting colour helped patients notice where, for example, the door to the bathroom was located and the passageway to the corridor. At SU1 and SU2, patients were guided to and from their room, with room numbers both on top of the door and on the wall close to the door to the room. At SU1, several so-called open workplaces in the corridor helped patients to find their way by highlighted coloured pillars next to the workplaces.’ **Anaker, von Koch**^17^
	*Lack of opportunities or incentives to be active outside of therapist-supervised time* (negative). Number of studies: 10 Opportunities often referred to organised activities with staff support, as well as access to space or equipment. There was a preference for these activities to be meaningful or enjoyable.	‘Participants also commented on the lack of therapy input on weekends which perpetuated a cycle of inactivity and boredom since there was no other “stimulation”. It was commonly acknowledged that the amount of time spent alone was perceived to be greater on weekend greater than on weekdays. “On the weekends … people do tend to get bored”. (P 6)’ **White, Alborough**^31^
	*The perceived and actual rules and culture of the ward* (negative). Number of studies: 9 This theme often describes a risk-averse culture around mobility and falls. Patients’ perceptions of ward rules influenced their physical activity, sometimes leading to feelings of ‘institutionalisation’. There is a strong link to the themes of institutionalisation, loss of autonomy, and a passive rehabilitation culture.	‘One factor that reduced motivation of staff to encourage stroke survivors to increase the amount of time they spend in standing and movement activity was concerned with fall prevention. In both inpatient settings, there was a lot of information displayed in staff and patient areas about preventing falls. The inpatient culture was very much directed toward reducing falls, with the primary way of doing this being to encourage patients to remain seated or in bed.’ **Morton, Hall**^26^
	*Institutionalisation, a loss of autonomy and a passive rehabilitation culture* (negative). Number of studies: 6 This theme highlights a lack of patient-initiated activity, low motivation, and a loss of control over daily life, often due to physical limitations from the stroke. Care processes and staff behaviour further contributed to a sense of institutionalisation. When staff were not involved in rehabilitation, patients felt time was ‘wasted’ or spent ‘waiting,’ especially in the evenings and weekends. Low activity was worsened when patients had to adjust to staff routines, worsened by poor punctuality.	‘In addition, this waiting to “be shunted around all day” was thought to establish a sense of “not knowing what they were doing” and a mindset that they had no control over their own daily schedule. This mindset was seen to extend over to the time outside of therapy, which was perceived to have a negative impact on their ability to drive their own recovery. “They say … How can I help with my recovery when I’m at everyone's mercy to tell me if or when?”’ **Eng, Brauer**^20^
COM-B: Social opportunity		
*Social influences* (Those interpersonal processes that can cause individuals to change their thoughts, feelings, or behaviours)	*Activity influenced by family and friends* (positive + negative). Number of studies: 10 Family and visitors often provided physical assistance and motivation for patients to be active. While generally positive, two studies noted a negative impact when families were overprotective and completed tasks for patients. Though it could be coded under social roles and identity, this theme was seen as more about social opportunity than motivation.	‘The arrival of their family and friends to the rehabilitation unit were reported by the majority of stroke survivors as an important facilitator of activity, not just physical activity (ie. walking), but socialising and engaging in leisure based activities. These visitors were important enablers of mobility for those dependent on others, with many not ‘going anywhere’ apart from the days when their family visited.’ **Janssen, Bird**^21^
	*Social activities or opportunities that provide a reason to be physically active* (positive). Number of studies: 8 Patients and staff viewed social and communal activities as motivating physical activity. The theme may be more common, but we included only extracts clearly related to physical, not social, activity.	‘“I took my friend there [the walker], and then I went to watch TV. […] I didn't walk very often, it was mostly to the TV and back, and then you meet some people. […] Because you get quite lonely, as people do when they are in a different environment.” Lars, age 84 years’ **Reinholdsson, Herranen**^35^
	*Staff interactions encouraging or discouraging physical activity* (positive and negative). Number of studies: 8 Staff attitudes and interactions with patients were seen to both positively and negatively affect motivation for physical activity.	‘We saw very little evidence of personal and social interaction between staff and patients, or between patients; when this did happen, patients commented on how much it inspired or encouraged them to do more.’ ***Jones NIHRJ 2020***
	*Patient interactions encouraging physical activity* (positive). Number of studies: 6 Peer support and seeing other patients’ progress were reported to positively impact physical activity.	‘Interacting with other stroke survivors was a strong source of motivation and hope for many interviewed. It provided reinforcement of the potential outcomes of putting in hard work to drive their own recovery, which they often acknowledged would require independent practice outside of therapy.’ **Eng, Brauer**^20^
COM-B: Physical capability		
*Skills* (An ability or proficiency acquired through practice).	*Physical impairments* (negative). Number of studies: 9 Physical impairments limiting the capability of patients to exercise or be physically active independently.	‘Physical impairments were perceived to be a powerful determinant of autonomous, self-initiated activity. Stroke survivors who were not independently mobile were potentially at risk of physical, cognitive, and social inactivity: “…any patient that is not mobile, without a family member present would pretty much always be either sitting in their room or on their bed in their room watching TV.” (P9) Occupational Therapist, 5.5 years on unit.’ **Janssen, Bird**^22^
COM-B: Physical and psychological capability		
*Skills*	*Fatigue and the need for rest* (negative). Number of studies: 8 Whilst fatigue did not align perfectly with the Theoretical Domains Framework definition of ‘Skills’, it was classified under the COM-B model's ‘Skills’ domain, as it relates to physical or psychological capability (i.e., stamina needed for the behaviour). Fatigue was reported as a result of stroke, stress, and inactivity, and in the context of needing rest after exertion. Patients mainly reported this, but staff were also hesitant to encourage physical activity in fatigued patients.	‘Participants also described that therapy was not the only contributor to feelings of exhaustion and tiredness but that recovering and adapting to physical limitations as a result of the stroke were physically, emotionally, and mentally demanding: “I tell them that the stroke – you get very tired. I yawn and carry on. I’ve never yawned so much in my life as I do since I’ve been here.” (Colleen)’ **Purcell, Scott**^27^
	*Importance of staff training and development* (positive and negative). Number of studies: 3 Staff development and training required to provide the skills to deliver stroke rehabilitation.	‘The Q study also identified issues that potentially will damage any recent gains made in RNs and HCAs rehabilitation knowledge and skills. There was strong agreement that RNs and HCAs need additional stroke specific training to integrate rehabilitation principles in care. Reliance on informal unit-based training is a high-risk strategy; participants in this study stated time and opportunity for such training is no longer available.’ **Clarke and Holt**^18^
COM-B: Psychological capability		
*Skills*	*Cognitive impairment* (negative). Number of studies: 6 cognitive impairments, whether real or perceived, that limit patients’ ability to exercise independently seem to influence staff behaviour towards physical activity. (Related to the Memory, Attention, and Decision Process domain).	‘Cognitive impairments were perceived as a barrier to all types of activity: …the patients who I think are here more for cognitive impairments …end up just not doing very much, being bored, lying in bed. (P20) Physiotherapist, 5 months on unit.’ **Janssen, Bird**^22^

Of the eight Theoretical Domains Framework domains, ‘Environmental Context and Resources’, defined as any circumstance of a person's situation or environment that discourages or encourages the development of skills and abilities, independence, social competence, and adaptive behaviour,^11^ was the most consistently recognised behavioural influence (16 of 17 studies). We identified five different themes within this domain, including four in at least nine different studies.

The theme of the availability of sufficient skilled staff to support physical activity largely related to the perception (of patients, families, and researchers) that staff were often too busy to assist with additional physical activity outside of basic needs and therapist-led rehabilitation. This issue particularly affected patients with more severe physical impairments (i.e., those needing assistance to mobilise), especially during evenings and weekends:

In many cases, participants expressed that they were too busy to prioritise facilitating patient access to EE [Enriched Environment] over their other demands. This was despite concerns that patients were often sedentary. (White, Alborough)^31^

Some studies also highlighted differences in staff ability, with less experienced staff lacking the knowledge and skills to support physical activity within their routines:

This factor suggests less-experienced staff endorse the rehabilitation philosophy expressed in stroke units, but these inexperienced nurses and therapists may lack of confidence in RNs’ and HCAs’ [Health Care Assistants’] skills to independently support ADL [activities of daily living] practice. (Clarke and Holt)^18^

This overlaps with the Theoretical Domains Framework domain of ‘Social or professional role or identity’, where other staff responsibilities often took precedence over facilitating activity:

Therapists reported a need to prioritise tasks that facilitated discharge and impacted on their ability to coach and train stroke survivors to practice with less supervision. (Stewart, Power)^30^

The theme of the design and use of the physical environment referred to positive and negative influences on physical activity. It appeared particularly relevant to people with cognitive impairment. Design features were noted to affect how people were able to navigate their environment:

…. I was lost every day. Everything looked the same. As soon as I walked out of my room the passages are all painted the same, the doors are the same. I’d go down to the dining room and I couldn’t ﬁnd my way back to my room. (P2, male, 76 yr)’ (Janssen, Bird)^21^

Spaces away from beds, such as dining rooms or gyms, provided opportunities for physical activity:

Communal mealtimes were considered to enhance frequent physical activity, for example, walking to and from meals and sitting up for breakfast and lunch. (Rosbergen, Brauer)^28^

However, different settings had varying space constraints, and even when space was available, it wasn’t always accessible for patients due to competing staff use:

Site 1 had no shared space (day room) in which patients and visitors could meet, and visitors were cramped by the bedside. Site 2 had a day room but it was used mainly for staff meetings and equipment storage; this was replicated at site 3, which had a garden but it was accessible only through the day room, which patients did not use. At site 4, the day room had become a storage area for wheelchairs and specialist stroke chairs; another seating area was routinely used by staff for taking breaks and storing cleaning equipment. (Jones, Gombert-Waldron)^23^

The theme of the lack of opportunities or incentives, passivity and institutionalisation referred to patients having little reason to be active outside of therapist-led sessions or basic needs like toileting. This issue was more prominent during evenings and weekends:

Participants also commented on the lack of therapy input on weekends which perpetuated a cycle of inactivity and boredom since there was no other ‘stimulation’. It was commonly acknowledged that the amount of time spent alone was perceived to be greater on weekend greater [sic] than on weekdays.

‘On the weekends … people do tend to get bored’. (P 6) (White, Alborough)^31^

Boredom and a sense of wasted time in rehabilitation settings were commonly reported:

Whilst many participants valued opportunities for rest, for others there was just too much downtime in their therapy schedule which contributed to boredom and frustration that there was ‘nothing to do’ and they were ‘wasting time’ in rehabilitation. (Kenah, Tavener)^32^

The reliance on staff to provide opportunities for activity contributed to a passive rehabilitation culture:

Similarly, while patients reportedly appreciated therapists’ encouragement to exercise on their own, it was rare to see them doing so. (Costa, Jones)^19^

The need to fit into staff routines, particularly for those with greater physical disability, reduced autonomy. Patients also felt they had little control over their rehabilitation programmes, which reinforced passivity:

In addition, this waiting to ‘be shunted around all day’ was thought to establish a sense of ‘not knowing what they were doing’ and a mindset that they had no control over their own daily schedule. This mindset was seen to extend over to the time outside of therapy, which was perceived to have a negative impact on their ability to drive their own recovery. ‘They say … How can I help with my recovery when I’m at everyone's mercy to tell me if or when?’ (Eng, Brauer)^20^

The theme of perceived and actual ward rules often reflected a risk-averse culture, particularly regarding falls. Patients frequently reported that they felt restricted from engaging in activities like independent walking:

In hospital, participants consistently reported a strong focus on safety from staff, particularly relating to falls. Many considered that standing and walking were discouraged to reduce this risk. (Simpson, Jose)^29^

This ‘paternalistic culture’, though understood by patients, staff, and families as well-intentioned, contributed to a sense of institutionalisation:

Engagement in activity was at times inﬂuenced by perception of what participants were and were not allowed to do, the perceived and actual ward rules reported to render them powerless to initiate activity autonomously. The perception that the rehabilitation staff needed to know where they were at all times was commonly reported with an awareness to ensure they adhered to any restrictions around independent mobility.

….I didn’t like to leave the room in case they said, ‘Well where is she? Where's she gone?’ So I just sat there and did a crossword puzzle and did that sort of thing. (P1, female, 90 yr) (Janssen, Bird)^21^

The Theoretical Domains Framework domain ‘Skills’ refers to abilities acquired through practice and is linked to the capability component of the COM-B model. This domain appeared in 12 studies, with four themes identified, most notably ‘physical impairments’, which was present in nine studies.

Physical impairments consistently limited patients’ ability to be physically active, particularly in terms of independent mobilisation and reliance on others for assistance. These impairments were largely attributed to the stroke:

Participants had wide ranging views on what could be offered to enhance engagement in occupations outside of therapy. Many understood the challenge in achieving this due to a combination of factors surrounding the person, the environment, and the occupation of choice. For example, Mark stated that although there may be many activities that stroke survivors may want to engage in outside of therapy, physical limitations may not make it possible ‘You’ve got to be well enough to be able to be mobile, to get out there, and do something. Some of the people there are not mobile at all. They’re just stuck in bed.’ (Purcell, Scott)^27^

The Theoretical Domains Framework domain ‘Social Influences’ refers to interpersonal processes that affect individuals’ thoughts, feelings, or behaviours and is linked to the social opportunity component of the COM-B model. We identified this domain in 11 studies, with ‘activity influenced by family and friends’ being the most frequent theme, appearing in 10 studies.

Family and friends were often facilitators of physical activity, providing assistance and motivation for walking and exercises. Patients with supportive visitors had more opportunities to stay active:

Family and visitors encouraged physical activity in hospital by practising walking, exercises and facilitating community visits. In contrast, people without family or with few visitors lacked such opportunities to be more active in hospital.

I had probably three a week [visitors]. They’re all much older than me…they’ve got problems and they couldn’t stay for long…I was available, all bushy-tailed, wide-eyed and waiting [to go for a walk], and it just didn’t always happen [Mary, (60–80y) living alone] (Simpson, Jose)^29^

Besides providing physical assistance, family and friends also helped overcome ward rules that restricted mobility:

Along with the need to conform to the routines and rules of the hospital environment, a loss of independence with mobility commonly limited stroke survivors from being able to freely leave their bedroom and participate in meaningful activities to occupy their non-therapy time.

‘I kept to the room; I didn’t walk around much on my own. Actually, the greater part of my stay I was not allowed to walk on my own, I only went for walks when my husband came to visit…so I was pretty much restricted until he came in.’ (P23, female, 68 y, moderate stroke) (Kenah, Tavener)^32^

## Discussion

We identified many positive and negative influences on physical activity outside of staff-led rehabilitation sessions of individuals hospitalised after a stroke. The review highlights the complexity of the behaviour and the challenges in trying to modify levels of physical activity and supports evidence of the benefit of multi-component interventions, targeting multiple behavioural determinants in improving levels of physical activity in this population.^37^ Although the most frequently highlighted influences on physical activity referred to three Theoretical Domains Framework domains: Environmental Context and Resources, Skills, and Social Influences, themes were identified in a total of eight domains, with overlap into others.

The most frequently recognised theme was the availability of sufficient skilled staff to facilitate physical activity. This theme, along with the lack of opportunities or incentives to be active outside of therapist-supervised time, was especially evident on weekends when fewer therapists are typically present. These themes are indirectly supported by quantitative evidence from several studies showing lower physical activity levels on weekends compared to weekdays.^38–44^

The theme of perceived and actual ward rules and culture often referred to a risk-averse approach that limits independent physical activity to reduce falls, which can cause serious physical and psychological harm.^45^ Many stroke patients in the hospital are at high risk of falling and may not fully appreciate or adjust to this risk.^46^ However, falls prevention strategies may not only be ineffective but can also restrict physical activity and overall autonomy.^47,48^ This may worsen issues like institutionalisation, loss of autonomy, and a passive rehabilitation culture.

The second most common theme was the design and use of the physical environment. The validity of the theme is supported by studies showing that design elements like single rooms versus multi-bed bays are linked to activity levels.^17,49^ Similarly, communal activities, such as shared meals, are also associated with increased physical activity.^50^

The availability and involvement of family and visitors can reduce reliance on staff for facilitating physical activity. Research supports this, showing family presence is linked to increased activity.^51,52^ However, this influence is complex. As seen in the studies reviewed^25,29^ and other work, family involvement can both help and hinder rehabilitation.^53^ While many families wish to be involved in their relative's recovery,^54,55^ this can strain relationships.^54^ Given the stress on relatives,^55,56^ more research is needed to find ways to better support and include them in rehabilitation. Additionally, social frailty may worsen health inequalities, requiring consideration of equitable rehabilitation support for patients without visitors to assist with physical activity.

The link between physical and functional impairments and activity levels after stroke is well established.^41,44,52,57–69^ This issue, largely related to capability, is hard to separate from the environmental and social factors discussed earlier. Evidence on improving physical activity post-stroke primarily focuses on patients with mild symptoms, with limited research on those with severe impairments, cognitive deficits, or post-stroke fatigue.^37^ This highlights the significant role of the COM-B model's capability category in determining activity levels after stroke.

A key limitation of the evidence in this review is the difficulty in consistently distinguishing between physical, social, and cognitive activity. While we focused on physical activity, it is possible that not all data were correctly classified. Similarly, while the review excluded data related to staff-led rehabilitation sessions, distinguishing between these and other activities was not always clear due to the nature of the data and analysis methods used in the primary research. As a result, some themes may have been unintentionally influenced by data or interpretations from staff-led sessions.

The synthesis of qualitative research has limitations, as we rely on the detail, context, and interpretation provided in the included manuscripts without access to the original data. Some nuances may have been missed or misinterpreted, leading to decontextualised findings.^70^ We took a pragmatic approach by focusing on the most frequently identified themes, though these may not represent the most influential factors and should not be viewed in isolation.

Intervention development should account for all identified factors. Although not highlighted in this review, complex micropolitics within healthcare teams^71^ may have shaped the behavioural influences and efforts to address them. Addressing these critical contextual factors is likely crucial for the success of future quality improvement or research initiatives.^71^

We used the COM-B and Theoretical Domains Framework to synthesise current evidence on influences affecting patient physical activity after stroke outside of staff-led rehabilitation sessions. Identified influences span all COM-B components and at least eight Theoretical Domains Framework domains. This review provides a theoretical foundation for future complex interventions and may aid the understanding of the barriers and facilitators to increasing patient physical activity. Recognising the complexity and interconnectedness of these factors may lead to more effective approaches for improving activity levels.

## Supplemental Material

sj-docx-1-cre-10.1177_02692155241293269 - Supplemental material for Influences on the physical activity behaviour of inpatients after stroke outside of staff-led rehabilitation sessions: a systematic review Supplemental material, sj-docx-1-cre-10.1177_02692155241293269 for Influences on the physical activity behaviour of inpatients after stroke outside of staff-led rehabilitation sessions: a systematic review by Peter Hartley, Katie Bond, Rachel Dance, Isla Kuhn, Joanne McPeake and Faye Forsyth in Clinical Rehabilitation

sj-docx-2-cre-10.1177_02692155241293269 - Supplemental material for Influences on the physical activity behaviour of inpatients after stroke outside of staff-led rehabilitation sessions: a systematic review Supplemental material, sj-docx-2-cre-10.1177_02692155241293269 for Influences on the physical activity behaviour of inpatients after stroke outside of staff-led rehabilitation sessions: a systematic review by Peter Hartley, Katie Bond, Rachel Dance, Isla Kuhn, Joanne McPeake and Faye Forsyth in Clinical Rehabilitation

## References

[1] Klassen TD Dukelow SP Bayley MT , et al. Higher doses improve walking recovery during stroke inpatient rehabilitation. Stroke 2020; 51: 2639–2648. 32811378 10.1161/STROKEAHA.120.029245

[2] Lohse KR Lang CE Boyd LA . Is more better? Using metadata to explore dose-response relationships in stroke rehabilitation. Stroke 2014; 45: 2053–2058. 24867924 10.1161/STROKEAHA.114.004695 PMC4071164

[3] Intercollegiate Stroke Working Party. National clinical guideline for stroke for the UK and Ireland. London, www.strokeguideline.org (2023).

[4] West T Bernhardt J . Physical activity in hospitalised stroke patients. Stroke Res Treat 2012; 2012: 813765. 21966599 10.1155/2012/813765 PMC3182066

[5] Jones F Gombert K Honey S , et al. Addressing inactivity after stroke: the Collaborative Rehabilitation in Acute Stroke (CREATE) study. Int J Stroke 2021; 16: 669–682. 33138735 10.1177/1747493020969367 PMC8366168

[6] Janssen J Klassen TD Connell LA , et al. Factors influencing the delivery of intensive rehabilitation in stroke: patient perceptions versus rehabilitation therapist perceptions. Phys Ther 2020; 100: 307–316. 31711211 10.1093/ptj/pzz159 PMC7246073

[7] Dorsch AK Thomas S Xu X , et al. SIRRACT: an international randomized clinical trial of activity feedback during inpatient stroke rehabilitation enabled by wireless sensing. Neurorehabil Neural Repair 2015; 29: 407–415. 25261154 10.1177/1545968314550369 PMC4375021

[8] O'Cathain A Croot L Duncan E , et al. Guidance on how to develop complex interventions to improve health and healthcare. BMJ Open 2019; 9: e029954. 10.1136/bmjopen-2019-029954 PMC6701588 31420394

[9] Michie S van Stralen MM West R . The behaviour change wheel: a new method for characterising and designing behaviour change interventions. Implement Sci 2011; 6: 42. 21513547 10.1186/1748-5908-6-42 PMC3096582

[10] Michie S Johnston M Abraham C , et al. Making psychological theory useful for implementing evidence based practice: a consensus approach. Qual Saf Health Care 2005; 14: 26–33. 15692000 10.1136/qshc.2004.011155 PMC1743963

[11] Cane J O'Connor D Michie S . Validation of the theoretical domains framework for use in behaviour change and implementation research. Implement Sci 2012; 7: 37. 22530986 10.1186/1748-5908-7-37 PMC3483008

[12] Michie S Atkins L West R . The behaviour change wheel: A guide to designing interventions. London: Silverback Publishing, 2014.

[13] Page MJ Moher D Bossuyt PM , et al. PRISMA 2020 Explanation and elaboration: updated guidance and exemplars for reporting systematic reviews. Br Med J 2021; 372: n160. 10.1136/bmj.n160 PMC8005925 33781993

[14] World Health Organisation. WHO guidelines on physical activity and sedentary behaviour. Geneva: World Health Organization, 2020.

[15] Hong QN Fàbregues S Bartlett G , et al. The Mixed Methods Appraisal Tool (MMAT) version 2018 for information professionals and researchers. Educ Inf 2018; 34: 285–291.

[16] Braun V Clarke V . Using thematic analysis in psychology. Qual Res Psychol 2006; 3: 77–101.

[17] Anaker A von Koch L Sjostrand C , et al. The physical environment and patients’ activities and care: A comparative case study at three newly built stroke units. J Adv Nurs 2018; 74: 1919–1931. 10.1111/jan.13690 29676493

[18] Clarke DJ Holt J . Understanding nursing practice in stroke units: a Q-methodological study. Disabil Rehabil 2015; 37: 1870–1880. 25412737 10.3109/09638288.2014.986588

[19] Costa A Jones F Kulnik ST , et al. Doing nothing? An ethnography of patients’ (In)activity on an acute stroke unit. Health (London) 2022; 26: 457–474. 33426969 10.1177/1363459320969784 PMC9163771

[20] Eng XW Brauer SG Kuys SS , et al. Factors affecting the ability of the stroke survivor to drive their own recovery outside of therapy during inpatient stroke rehabilitation. Stroke Res Treat 2014; 2014: 626538. 24800104 10.1155/2014/626538 PMC3985302

[21] Janssen H Bird ML Luker J , et al. Stroke survivors’ perceptions of the factors that influence engagement in activity outside dedicated therapy sessions in a rehabilitation unit: a qualitative study. Clin Rehabil 2022; 36: 822–830. 35290136 10.1177/02692155221087424

[22] Janssen H Bird ML Luker J , et al. Impairments, and physical design and culture of a rehabilitation unit influence stroke survivor activity: qualitative analysis of rehabilitation staff perceptions. Disabil Rehabil 2022; 44: 8436–8441. 35113761 10.1080/09638288.2021.2019840

[23] Jones F Gombert-Waldron K Honey S , et al. Using co-production to increase activity in acute stroke units: the CREATE mixed-methods study. Health Serv Deliv Res 2020; 8. 32894667

[24] Loft MI Martinsen B Esbensen BA , et al. Call for human contact and support: an interview study exploring patients’ experiences with inpatient stroke rehabilitation and their perception of nurses’ and nurse assistants’ roles and functions. Disabil Rehabil 2019; 41: 396–404. 29065725 10.1080/09638288.2017.1393698

[25] Maclean N Pound P Wolfe C , et al. Qualitative analysis of stroke patients’ motivation for rehabilitation. Br Med J 2000; 321: 1051–1054. 11053175 10.1136/bmj.321.7268.1051 PMC27512

[26] Morton S Hall J Fitzsimons C , et al. A qualitative study of sedentary behaviours in stroke survivors: non-participant observations and interviews with stroke service staff in stroke units and community services. Disabil Rehabil 2022; 44: 5964–5973. 34304649 10.1080/09638288.2021.1955307

[27] Purcell S Scott P Gustafsson L , et al. Stroke survivors’ experiences of occupation in hospital-based stroke rehabilitation: a qualitative exploration. Disabil Rehabil 2020; 42: 1880–1885. 30672347 10.1080/09638288.2018.1542460

[28] Rosbergen ICM Brauer SG Fitzhenry S , et al. Qualitative investigation of the perceptions and experiences of nursing and allied health professionals involved in the implementation of an enriched environment in an Australian acute stroke unit. BMJ Open 2017; 7: e018226. 10.1136/bmjopen-2017-018226 PMC5778299 29273658

[29] Simpson DB Jose K English C , et al. Factors influencing sedentary time and physical activity early after stroke: a qualitative study. Disabil Rehabil 2022; 44: 3501–3509. 33399023 10.1080/09638288.2020.1867656

[30] Stewart C Power E McCluskey A , et al. Development of a participatory, tailored behaviour change intervention to increase active practice during inpatient stroke rehabilitation. Disabil Rehabil 2020; 42: 3516–3524. 30982361 10.1080/09638288.2019.1597178

[31] White JH Alborough K Janssen H , et al. Exploring staff experience of an “enriched environment” within stroke rehabilitation: a qualitative sub-study. Disabil Rehabil 2014; 36: 1783–1789. 24369101 10.3109/09638288.2013.872200

[32] Kenah K Tavener M Bernhardt J , et al. “Wasting time”: a qualitative study of stroke survivors’ experiences of boredom in non-therapy time during inpatient rehabilitation. Disabil Rehabil 2024; 46: 2799–2807. 37409578 10.1080/09638288.2023.2230131

[33] Lipson-Smith R Zeeman H Muns L , et al. The role of the physical environment in stroke recovery: evidence-based design principles from a mixed-methods multiple case study. PLoS One 2023; 18: e0280690. 10.1371/journal.pone.0280690 PMC10256226 37294748

[34] White JH Bartley E Janssen H , et al. Exploring stroke survivor experience of participation in an enriched environment: a qualitative study. Disabil Rehabil 2015; 37: 593–600. 25754445 10.3109/09638288.2014.935876

[35] Reinholdsson M Herranen G Sunnerhagen KS , et al. Patient experiences of physical activity and inactivity in the stroke unit: an interview study. J Rehabil Med 2024; 56: jrm19502. 10.2340/jrm.v56.19502 PMC10865105 38329296

[36] Donetto S Jones F Clarke DJ , et al. Exploring liminality in the co-design of rehabilitation environments: the case of one acute stroke unit. Health Place 2021; 72: 102695. 34768039 10.1016/j.healthplace.2021.102695 PMC8633757

[37] Paterson S Dawes H Winward C , et al. Use of the capability, opportunity and motivation behaviour model (COM-B) to understand interventions to support physical activity behaviour in people with stroke: an overview of reviews. Clin Rehabil 2024; 38: 543–557. 38192225 10.1177/02692155231224365 PMC10898199

[38] Alt Murphy M Andersson S Danielsson A , et al. Comparison of accelerometer-based arm, leg and trunk activity at weekdays and weekends during subacute inpatient rehabilitation after stroke. J Rehabil Med 2019; 51: 426–433. 30951177 10.2340/16501977-2553

[39] de Jong AU Smith M Callisaya ML , et al. Sedentary time and physical activity patterns of stroke survivors during the inpatient rehabilitation week. Int J Rehabil Res 2021; 44: 131–137. 33724969 10.1097/MRR.0000000000000461

[40] Gustafsson L McKenna K . Is there a role for meaningful activity in stroke rehabilitation? Top Stroke Rehabil 2010; 17: 108–118. 20542853 10.1310/tsr1702-108

[41] Janssen H Ada L Bernhardt J , et al. Physical, cognitive and social activity levels of stroke patients undergoing rehabilitation within a mixed rehabilitation unit. Clin Rehabil 2014; 28: 91–101. 23193176 10.1177/0269215512466252

[42] Mackey F Ada L Heard R , et al. Stroke rehabilitation: are highly structured units more conducive to physical activity than less structured units? Arch Phys Med Rehabil 1996; 77: 1066–1070. 8857888 10.1016/s0003-9993(96)90070-2

[43] Barrett M Snow JC Kirkland MC , et al. Excessive sedentary time during in-patient stroke rehabilitation. Top Stroke Rehabil 2018; 25: 366–374. 29609499 10.1080/10749357.2018.1458461

[44] Selenitsch NA Gill SD . Stroke survivor activity during subacute inpatient rehabilitation: how active are patients? Int J Rehabil Res 2019; 42: 82–84. 30379697 10.1097/MRR.0000000000000326

[45] Montero-Odasso M van der Velde N Martin FC , et al. World guidelines for falls prevention and management for older adults: a global initiative. Age Ageing 2022; 51: afac205. 10.1093/ageing/afac205 PMC9523684 36178003

[46] Aihara S Kitamura S Dogan M , et al. Patients’ thoughts on their falls in a rehabilitation hospital: a qualitative study of patients with stroke. BMC Geriatr 2021; 21: 713. 34922484 10.1186/s12877-021-02649-1 PMC8684226

[47] Growdon ME Shorr RI Inouye SK . The tension between promoting mobility and preventing falls in the hospital. JAMA Intern Med 2017; 177: 759–760. 28437517 10.1001/jamainternmed.2017.0840 PMC5500203

[48] LeLaurin JH Shorr RI . Preventing falls in hospitalized patients: state of the science. Clin Geriatr Med 2019; 35: 273–283. 30929888 10.1016/j.cger.2019.01.007 PMC6446937

[49] Anaker A von Koch L Sjostrand C , et al. A comparative study of patients’ activities and interactions in a stroke unit before and after reconstruction-the significance of the built environment. PLoS One 2017; 12: e0177477. 10.1371/journal.pone.0177477 PMC5519004 28727727

[50] Hokstad A Indredavik B Bernhardt J , et al. Hospital differences in motor activity early after stroke: a comparison of 11 Norwegian stroke units. J Stroke Cerebrovasc Dis 2015; 24: 1333–1340. 25906937 10.1016/j.jstrokecerebrovasdis.2015.02.009

[51] Prakash V Shah MA Hariohm K . Family's presence associated with increased physical activity in patients with acute stroke: an observational study. Braz J Phys Ther 2016; 20: 306–311. 27556386 10.1590/bjpt-rbf.2014.0172 PMC5015679

[52] Skarin M Sjoholm A Nilsson A , et al. A mapping study on physical activity in stroke rehabilitation: establishing the baseline. J Rehabil Med 2013; 45: 997–1003. 24150662 10.2340/16501977-1214

[53] Bezmez D Shakespeare T Yardimci S . Family role in in-patient rehabilitation: the cases of England and Turkey. Disabil Rehabil 2021; 43: 559–567. 31257955 10.1080/09638288.2019.1632941

[54] Lobo EH Frolich A Abdelrazek M , et al. Information, involvement, self-care and support-the needs of caregivers of people with stroke: a grounded theory approach. PLoS One 2023; 18: e0281198. 10.1371/journal.pone.0281198 PMC9888718 36719929

[55] Luker J Murray C Lynch E , et al. Carers’ experiences, needs, and preferences during inpatient stroke rehabilitation: a systematic review of qualitative studies. Arch Phys Med Rehabil 2017; 98: 1852–1862.e13. 28363703 10.1016/j.apmr.2017.02.024

[56] Young ME Lutz BJ Creasy KR , et al. A comprehensive assessment of family caregivers of stroke survivors during inpatient rehabilitation. Disabil Rehabil 2014; 36: 1892–1902. 24467676 10.3109/09638288.2014.881565 PMC4959419

[57] Bernhardt J Chan J Nicola I , et al. Little therapy, little physical activity: rehabilitation within the first 14 days of organized stroke unit care. J Rehabil Med 2007; 39: 43–48. 17225037 10.2340/16501977-0013

[58] Esmonde T McGinley J Wittwer J , et al. Stroke rehabilitation: patient activity during non-therapy time. Aust J Physiother 1997; 43: 43–51. 11676671 10.1016/s0004-9514(14)60401-3

[59] Henderson CE Fahey M Brazg G , et al. Predicting discharge walking function with high-intensity stepping training during inpatient rehabilitation in nonambulatory patients poststroke. Arch Phys Med Rehabil 2022; 103: S189–S196. 10.1016/j.apmr.2020.10.127 33227267

[60] Kanai M Nozoe M Ohtsubo T , et al. Relationship of Functional Outcome With Sarcopenia and Objectively Measured Physical Activity in Patients With Stroke Undergoing Rehabilitation. J Aging Phys Act 2023; 31: –6. 10.1123/japa.2022-0025 35461188

[61] Kubo H Kanai M Nozoe M , et al. Daily steps are associated with walking ability in hospitalized patients with sub-acute stroke. Sci Rep 2022; 12: 12217. 35843983 10.1038/s41598-022-16416-8 PMC9288997

[62] Kunkel D Fitton C Burnett M , et al. Physical inactivity post-stroke: a 3-year longitudinal study. Disabil Rehabil 2015; 37: 304–310. 24828312 10.3109/09638288.2014.918190

[63] Lacroix J Daviet JC Borel B , et al. Physical activity level among stroke patients hospitalized in a rehabilitation unit. PM R 2016; 8: 97–104. 26107540 10.1016/j.pmrj.2015.06.011

[64] Norvang OP Hokstad A Taraldsen K , et al. Time spent lying, sitting, and upright during hospitalization after stroke: a prospective observation study. BMC Neurol 2018; 18: 38. 30180819 10.1186/s12883-018-1134-0 PMC6122609

[65] Nozoe M Kubo H Furuichi A , et al. Physical activity, physical function, and quadriceps muscle thickness in male patients with sub-acute stroke during hospitalization: a pilot study. Eur Neurol 2019; 80: 157–162. 10.1159/000494991 30463057

[66] Shimizu N Hashidate H Ota T , et al. Characteristics of intensity-based physical activity according to gait ability in people hospitalized with subacute stroke: a cross-sectional study. Phys Ther Res 2019; 22: 17–25. 31289708 10.1298/ptr.E9971 PMC6599747

[67] Shimizu N Hashidate H Ota T , et al. Physical activity according to sit-to-stand, standing, and stand-to-sit abilities in subacute stroke with walking difficulty: a cross-sectional study. Physiother Theory Pract 2023; 39: 2327–2335. 35543544 10.1080/09593985.2022.2074928

[68] Yamada R Shimizu S Suzuki Y , et al. Factors related to daily step counts of stroke patients during hospitalization in a convalescent rehabilitation ward. J Stroke Cerebrovasc Dis 2022; 31: 106398. 35219974 10.1016/j.jstrokecerebrovasdis.2022.106398

[69] King A McCluskey A Schurr K . The time use and activity levels of inpatients in a co-located acute and rehabilitation stroke unit: an observational study. Top Stroke Rehabil 2011; 18: 654–665. 22120034 10.1310/tsr18s01-654

[70] Thomas J Harden A . Methods for the thematic synthesis of qualitative research in systematic reviews. BMC Med Res Methodol 2008; 8: 45. 18616818 10.1186/1471-2288-8-45 PMC2478656

[71] Rogers L De Brun A Birken SA , et al. The micropolitics of implementation; a qualitative study exploring the impact of power, authority, and influence when implementing change in healthcare teams. BMC Health Serv Res 2020; 20: 1059. 33228702 10.1186/s12913-020-05905-z PMC7684932

