# Supplementary material for: Influences on the physical activity behaviour of inpatients after stroke outside of staff-led rehabilitation sessions: a systematic review
Source: Clin Rehabil. 2024 Nov 10;39(1):109–27. doi: 10.1177/02692155241293269 (PMC11776358; doi:10.1177/02692155241293269)
Supplement: sj-docx-1-cre-10.1177_02692155241293269 - Supplemental material for Influences on the physical activity behaviour of inpatients after stroke outside of staff-led rehabilitation sessions: a systematic review [file sj-docx-1-cre-10.1177_02692155241293269.docx]

# **Supplementary Materials 1**

# Table 1.1. Search results

| **Database** | **Search results 6^th^ Dec 2022** | **Updated search results 16^th^ January 2024** | **Updated search results 2^nd^ October 2024** |
| --- | --- | --- | --- |
| Medline via Ovid | 964 | 111 | 68 |
| AMED via Ovid | 216 | 17 | 4* |
| CINAHL via EbscoHost | 276 | 50 | 9 |
| PsycInfo via EbscoHost | 108 | 18 | 2 |
| Total | 1456 | 196 | 84 |
| Total deduplicated | 1168 | 162 | 77 |

# Table 1.2. Medline search strategy

| **Step** | **Query** |
| --- | --- |
| 1. | (((rehabilitation or physical* or therap* or physiotherap* or practice or exercise*) adj4 (activ* or dose or intensity or frequency or amount or repetitions)) or (activ* adj4 (increase* or dose or intensity or frequency or amount or repetitions)) or ((unsupervised or supervised) adj3 practice) or meaningful activ* or sedent* or inactiv* or non-sedent*).ti,ab,kw,kf. |
| 2. | (Stroke or cva or cerebrovascular accident*).ti,ab,kw,kf. or exp stroke/ |
| 3. | (((Inpatient* or in-patient* or acute or stroke or rehabilitation) adj3 (unit* or hospital* or ward* or admitted or admission)) or "inpatient rehabilitation" or "in-patient rehabilitation").ab,ti,kw,kf. or exp Inpatients/ |
| 4. | 1 and 2 and 3 |

# Table 1.3. PsycInfo search strategy

| Step | Query |
| --- | --- |
| S1 | ti(((rehabilitation or physical* or therap* or physiotherap* or practice or exercise*) n4 (activ* or dose or intensity or frequency or amount or repetitions)) or (activ* n4 (increase* or dose or intensity or frequency or amount or repetitions)) or ((unsupervised or supervised) n3 practice) or "meaningful activ*" or sedent* or inactiv* or non-sedent*) or ab(((rehabilitation or physical* or therap* or physiotherap* or practice or exercise*) n4 (activ* or dose or intensity or frequency or amount or repetitions)) or (activ* n4 (increase* or dose or intensity or frequency or amount or repetitions)) or ((unsupervised or supervised) n3 practice) or "meaningful activ*" or sedent* or inactiv* or non-sedent*) |
| S2 | ti(Stroke or cva or "cerebrovascular accident*") or ab(Stroke or cva or "cerebrovascular accident*") |
| S3 | DE "Cerebrovascular Accidents" |
| S4 | S2 OR S3 |
| S5 | (((Inpatient* or in-patient* or acute or stroke or rehabilitation) n3 (unit* or hospital* or ward* or admitted or admission)) or "inpatient rehabilitation" or "in-patient rehabilitation") |
| S6 | DE "Hospitalized Patients" |
| S7 | S5 OR S6 |
| S8 | S1 AND S4 AND S7 |

# Table 1.4. CINAHL search strategy

| **Step** | **Query** |
| --- | --- |
| S1 | ti(((rehabilitation or physical* or therap* or physiotherap* or practice or exercise*) n4 (activ* or dose or intensity or frequency or amount or repetitions)) or (activ* n4 (increase* or dose or intensity or frequency or amount or repetitions)) or ((unsupervised or supervised) n3 practice) or "meaningful activ*" or sedent* or inactiv* or non-sedent*) or ab(((rehabilitation or physical* or therap* or physiotherap* or practice or exercise*) n4 (activ* or dose or intensity or frequency or amount or repetitions)) or (activ* n4 (increase* or dose or intensity or frequency or amount or repetitions)) or ((unsupervised or supervised) n3 practice) or "meaningful activ*" or sedent* or inactiv* or non-sedent*) |
| S2 | ti(Stroke or cva or "cerebrovascular accident*") or ab(Stroke or cva or "cerebrovascular accident*") |
| S3 | (MH "Stroke+") |
| S4 | S2 OR S3 |
| S5 | (((Inpatient* or in-patient* or acute or stroke or rehabilitation) n3 (unit* or hospital* or ward* or admitted or admission)) or "inpatient rehabilitation" or "in-patient rehabilitation") |
| S6 | (MH "Inpatients") |
| S7 | S5 OR S6 |
| S8 | S1 AND S4 AND S7 |

Table 1.5. AMED search strategy

| **Step** | **Query** |
| --- | --- |
| 1. | (((rehabilitation or physical* or therap* or physiotherap* or practice or exercise*) adj4 (activ* or dose or intensity or frequency or amount or repetitions)) or (activ* adj4 (increase* or dose or intensity or frequency or amount or repetitions)) or ((unsupervised or supervised) adj3 practice) or meaningful activ* or sedent* or inactiv* or non-sedent*).ti,ab. |
| 2. | (Stroke or cva or cerebrovascular accident*).ti,ab. |
| 3. | exp stroke/ |
| 4. | 2 or 3 |
| 5. | (((Inpatient* or in-patient* or acute or stroke or rehabilitation) adj3 (unit* or hospital* or ward* or admitted or admission)) or "inpatient rehabilitation" or "in-patient rehabilitation").ab,ti. |
| 6. | exp Inpatients/ |
| 7. | 5 or 6 |
| 8. | 1 and 4 and 7 |

# Mixed Methods Appraisal Tool (MMAT) Scoring

Table 2.1. Qualitative methodological criteria

| **Study** | **S1. Are there clear research questions?** | **S2. Do the collected data allow to address the research questions?** | **1.1. Is the qualitative approach appropriate to answer the research question?** | **1.2. Are the qualitative data collection methods adequate to address the research question?** | **1.3. Are the findings adequately derived from the data?** | **1.4. Is the interpretation of results sufficiently substantiated by data?** | **1.5. Is there coherence between qualitative data sources, collection, analysis and interpretation?** |
| --- | --- | --- | --- | --- | --- | --- | --- |
| Jones, Gombert ^5,^ Anaker, von Koch ^16^ | Yes | Yes | Yes | Yes | Yes | Yes | Yes |
| Clarke and Holt ^17^ | Yes | Yes | Yes | Yes | Yes | Yes | Yes |
| Eng, Brauer ^19^ | Yes | Yes | Yes | Yes | Yes | Can’t tell | Yes |
| Janssen, Bird ^20^ | Yes | Yes | Yes | Yes | Yes | Yes | Yes |
| Kenah, Tavener ^31^ | Yes | Yes | Yes | Yes | Yes | Yes | Yes |
| Janssen, Bird ^21^ | Yes | Yes | Yes | Yes | Yes | Yes | Yes |
| Jones, Gombert-Waldron ^22^ | Yes | Yes | Yes | Yes | Yes | Yes | Yes |
| Jones, Gombert-Waldron ^22^ | Yes | Yes | Yes | Yes | Yes | Yes | Yes |
| Costa, Jones ^18^ | Yes | Yes | Yes | Yes | Yes | Yes | Yes |
| Lipson-Smith, Zeeman ^32^ | Yes | Yes | Yes | Yes | Yes | Yes | Yes |
| Loft, Martinsen ^23^ | Yes | Yes | Yes | Yes | Yes | Yes | Yes |
| Maclean, Pound ^24^ | Yes | Yes | Yes | Yes | Can’t tell | Can’t tell | Can’t tell |
| Morton, Hall ^25^ | Yes | Yes | Yes | Yes | Yes | Yes | Yes |
| Purcell, Scott ^26^ | Yes | Yes | Yes | Yes | Yes | Yes | Yes |
| Reinholdsson, Herranen ^34^ | Yes | Yes | Yes | Yes | Yes | Can’t tell | Can’t tell |
| Rosbergen, Brauer ^27^ | Yes | Yes | Yes | Yes | Yes | Yes | Yes |
| Simpson, Jose ^28^ | Yes | Yes | Yes | Yes | Yes | Yes | Yes |
| Stewart, Power ^29^ | Yes | Yes | Yes | Yes | Yes | Yes | Yes |
| White, Alborough ^30^ | Yes | Yes | Yes | Yes | Yes | Yes | Yes |
| White, Bartley ^33^ | Yes | Yes | Yes | Yes | Yes | Yes | Yes |

Table 2.2. Mixed methods methodological criteria

| **Study** | **S1. Are there clear research questions?** | **S2. Do the collected data allow to address the research questions?** | **5.1. Is there an adequate rationale for using a mixed methods design to address the research question?** | **5.2. Are the different components of the study effectively integrated to answer the research question?** | **5.3. Are the outputs of the integration of qualitative and quantitative components adequately interpreted?** | **5.4. Are divergences and inconsistencies between quantitative and qualitative results adequately addressed?** | **5.5. Do the different components of the study adhere to the quality criteria of each tradition of the methods involved?** |
| --- | --- | --- | --- | --- | --- | --- | --- |
| Anaker, von Koch ^16^  Mixed-methods study | Yes | Yes | Yes | Yes | Yes | Yes | Yes |
| Clarke and Holt ^17^  Mixed-methods study | Yes | Yes | Yes | Yes | Yes | Yes | Yes |
| Lipson-Smith, Zeeman ^32^ | Yes | Yes | Yes | Yes | Yes | Yes | Yes |

Table 2.3. Quantitative methods methodological criteria

| **Study** | **S1. Are there clear research questions?** | **S2. Do the collected data allow to address the research questions?** | **4.1. Is the sampling strategy relevant to address the research question?** | **4.2. Is the sample representative of the target population?** | **4.3. Are the measurements appropriate?** | **4.4. Is the risk of nonresponse bias low?** | **4.5. Is the statistical analysis appropriate to answer the research question?** |
| --- | --- | --- | --- | --- | --- | --- | --- |
| Anaker, von Koch ^16^ | Yes | Yes | Yes | Can’t tell | Yes | Can’t tell | Yes |
| Clarke and Holt ^17^ | Yes | Yes | Yes | Yes | Yes | Can’t tell | Yes |
| Lipson-Smith, Zeeman ^32^ | Yes | Yes | Yes | Yes | Yes | Yes | Yes |
